# Abnormally high expression of CHI3L1 in peripheral blood mononuclear cells and serum and their potential diagnosis and prediction from lymphoma patients

**DOI:** 10.3389/fimmu.2025.1557802

**Published:** 2025-04-07

**Authors:** Chao Wang, Langui Tang, Haibing Luo, Juan Liang, Yanzhao Huang, Kaiyun Guo, Rong Liu, Yuxing He, Yan Gao, Ming Lei

**Affiliations:** ^1^ Changde Hospital, Xiangya School of Medicine, Central South University (The First People’s Hospital of Changde City), Changde, China; ^2^ Department of Clinical Laboratory, The Fourth People’s Hospital of Changde, Changde, China; ^3^ Hengyang Medical College, University of South China, Hengyang, China

**Keywords:** lymphoma, CHI3L1, bioinformatics, overall survival, prognosis

## Abstract

**Aim:**

This study aimed to investigate the expression of CHI3L1 in aggressive lymphomas and assess its potential as a diagnostic and prognostic biomarker.

**Methodology:**

This study investigates the expression of CHI3L1 protein in the peripheral blood of patients with aggressive lymphoma and healthy controls using enzyme-linked immunosorbent assay (ELISA). The prognostic significance of CHI3L1 was assessed through Cox regression and Kaplan-Meier survival analyses. The differences in CHI3L1 expression between lymphoma and control samples were analyzed using the lymphoma-related gene expression datasets GSE25638 and GSE56315, as well as their combined dataset (GSE25638 and GSE56315). Subsequently, a prognostic analysis of CHI3L1 was conducted using the lymphoma tissue sample gene expression dataset GSE31312. Weighted gene co-expression network analysis (WGCNA) identified genes co-expressed with CHI3L1, and a protein-protein interaction (PPI) network was constructed. RT-qPCR was used to further validate CHI3L1 expression in peripheral blood mononuclear cells (PBMCs) from lymphoma patients

**Results:**

The serum CHI3L1 protein expression in patients with aggressive lymphoma was significantly higher than that in healthy controls (p<0.001). Moreover, CHI3L1 levels were significantly elevated in stage III~IV patients compared to stage I~II patients (P = 0.001). One-way Cox regression and Kaplan-Meier analyses further demonstrated that high CHI3L1 expression was closely associated with shorter overall survival (p<0.001). Bioinformatics analysis revealed that CHI3L1 expression was significantly elevated in lymphoma samples compared to normal controls (p < 0.05), with diagnostic AUC values of 0.92, 0.99, and 0.93, indicating high diagnostic accuracy. Furthermore, patients with high CHI3L1 expression exhibited significantly shorter overall survival (p < 0.05), suggesting a potential association with poor prognosis. Co-expression analysis identified 605 genes associated with key biological processes, including the inflammatory response, signal transduction, and apoptosis. These genes were enriched in functional pathways such as mineral uptake and the Toll-like receptor signaling pathway. Validation experiments confirmed that CHI3L1 gene expression in PBMCs of patients with aggressive lymphoma was significantly higher than that in healthy individuals (p<0.01).

**Conclusion:**

This study demonstrates that elevated CHI3L1 expression is strongly associated with lymphoma onset, progression, severity, and poor prognosis, underscoring its potential as both a diagnostic and prognostic biomarker. Moreover, CHI3L1 may contribute to lymphoma progression by regulating key biological processes.

## 1 Introduction

Lymphoma is a malignancy which originates from the lymphatic system (1, 2), and of which incidence steadily increases in recent years (3). Due to the complexity of its pathological classification, lymphoma includes several subtypes which have distinct biological characteristics. Among these, aggressive lymphomas, particularly diffuse large B-cell lymphoma (DLBCL), are frequently associated with poor prognosis and a high risk of relapse due to their invasiveness and significant heterogeneity (4). Despite advances in chemotherapy, immunotherapy, and targeted therapies, some patients continue to exhibit resistance to current treatment options, which significantly limits therapeutic efficacy and complicates disease management (4, 5). Therefore, there is an urgent need to identify new biomarkers and therapeutic targets to optimize treatment strategies and improve patient outcomes.

CHI3L1 (Chitinase-3-like protein 1, YKL-40) is a glycoprotein derived from the 18th glycosyl hydrolase family. Despite lacking enzymatic activity, it plays a broad range of roles in various physiological and pathological processes (6, 7). CHI3L1 is primarily secreted by macrophages, fibroblasts, and tumor cells, and is involved in extracellular matrix remodeling, cell proliferation, angiogenesis, and regulation of inflammatory responses (6, 8–11). Recent studies have shown that CHI3L1 is highly expressed in solid tumors, including lung cancer, breast cancer, and colorectal cancer, and is closely associated with tumor invasiveness, chemotherapy resistance, and poor prognosis (12–18). In particular, in cancer-related inflammatory responses, CHI3L1 promotes tumor cell proliferation, migration, and immune evasion by binding to specific receptors (e.g., IL-13Rα2, CD44) and activating downstream signaling pathways (e.g., MAPK, PI3K/AKT) (18, 19). However, the role of CHI3L1 in hematologic malignancies remains insufficiently explored, particularly regarding its expression characteristics and prognostic value in aggressive lymphomas. With the widespread application of high-throughput sequencing technologies, bioinformatics analysis has become a crucial tool in tumor research. By utilizing large-scale multi-omics data from public databases such as TCGA and GEO, it is possible to comprehensively analyze the expression characteristics of candidate genes in tumors and their association with clinical features. Such analyses not only identify potential pathogenic genes but also provide a strong foundation for the screening and validation of biomarkers.

The innovation of this study lies in the first systematic evaluation and validation of the expression characteristics and prognostic value of CHI3L1 in aggressive lymphoma. Through bioinformatics analysis and experimental validation, this study comprehensively examines the potential of CHI3L1 as a biomarker for the progression and prognosis of aggressive lymphoma. The aim of our research mainly provides new theoretical insights and identify potential targets for understanding the molecular mechanisms of this disease and advancing clinical precision medicine, thereby offering scientific evidence to support future diagnostic and therapeutic strategies.

## 2 Materials and methods

### 2.1 Clinical information

**Figure 1:**
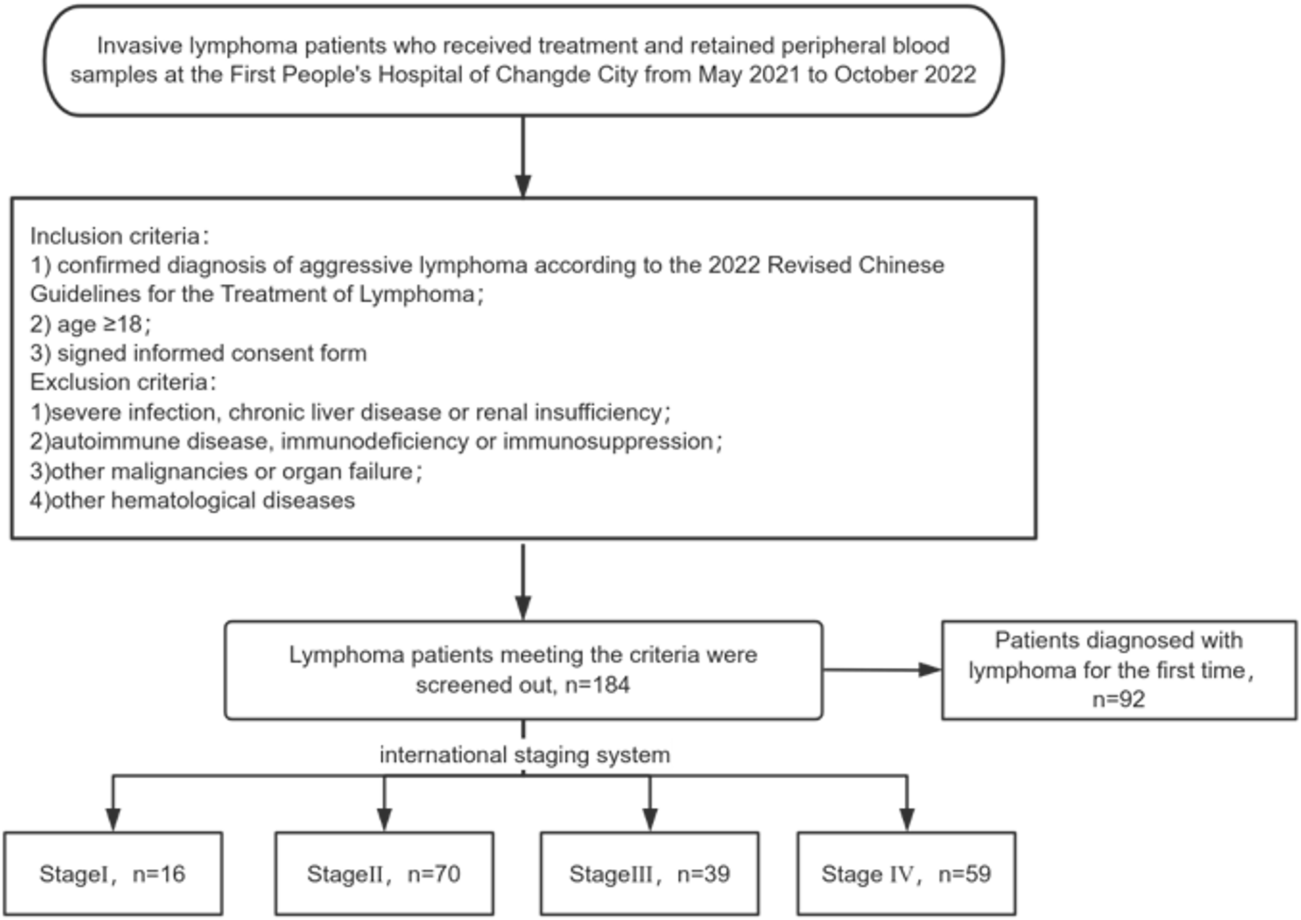
Flowchart of lymphoma patient screening.

In this study, patients with aggressive lymphoma who received treatment at the First People’s Hospital of Changde city from May 2021 to October 2022 and whose peripheral blood samples were retained from the biobank of the hospital were selected as the study objects. The inclusion criteria included (1) confirmed diagnosis of aggressive lymphoma according to the 2022 Revised Chinese Guidelines for the Treatment of Lymphoma, (2) age ≥18 years, and (3) signed an informed consent form. The exclusion criteria included (1) a combination of severe infection, chronic liver disease or renal insufficiency; (2) a combination of autoimmune disease, immunodeficiency or immunosuppression; (3) a combination of other malignancies or organ failure; and (4) a combination of other hematological diseases. Moreover, the peripheral blood samples of the control group were from healthy individuals (according to medical check-ups) with no family history of hematological diseases in the biobank of our hospital. This study was approved by the Medical Ethics Committee of the First People’s Hospital of Changde City. The flowchart of patient screening is shown in **Figure 1**.

### 2.2 Expression of the CHI3L1 protein in the serum of patients with aggressive lymphoma as determined by ELISA

The serum samples from 184 patients with aggressive lymphoma and 40 healthy controls stored in the biobank were thawed, and enzyme-linked immunosorbent assay (ELISA) was used to measure CHI3L1 levels in the serum. The assay process was carried out in accordance with the manufacturer’s instructions (Hangzhou Puwang Biotechnology Co., Ltd.).

### 2.3 Bioinformatics analysis

#### 2.3.1 Data acquisition and preprocessing

Lymphoma-related datasets (GSE25638, GSE56315, and GSE31312) were obtained from the Gene Expression Omnibus (GEO) of the Cancer Genome Atlas (https://www.ncbi.nlm.nih.gov/geoprofiles/). The GSE25638 dataset (based on the GPL570 platform) contains RNA expression profiling data from 13 normal B-cell samples and 84 lymphoma samples; the GSE56315 dataset (based on the GPL570 platform) contains expression profiling data from 33 normal B-cell samples and 55 lymphoma samples; and the GSE31312 dataset (based on the GPL570 platform) contains clinical information and transcription profile data from 498 tumor tissues. To increase the sample size to improve the reliability of the subsequent analyses, we merged the GSE25638 dataset and the GSE56315 dataset into one dataset for subsequent analyses.

#### 2.3.2 Differential CHI3L1 expression and prognostic analysis

With respect to the GSE25638 dataset, GSE56315 dataset and merged dataset, the Wilcoxon rank sum test was used to compare the differences in the expression of CHI3L1 between lymphoma tissues and normal B cells, and box plots were drawn. In addition, the diagnostic value of CHI3L1 for lymphoma was assessed via receiver operating characteristic (ROC) curve analysis, and the diagnostic efficacy was assessed via the area under the curve (AUC).

Based on the GSE31312 dataset, the patients were divided into high- and low-expression groups according to the optimal cutoff value of CHI3L1 expression, and the relationship between CHI3L1 expression and overall survival (OS) in lymphoma patients was evaluated via the Kaplan–Meier method and the log-rank test. Survival curves were obtained by using the ‘survival’ package of the R language. Moreover, ROC curves were plotted to analyze the ability of the CHI3L1 expression level to predict the survival status of lymphoma patients at 1, 3 and 5 years.

#### 2.3.3 Screening of genes associated with CHI3L1 expression in lymphoma

In this study, we first integrated the dataset and identified genes that were differentially expressed between lymphoma samples and normal B-cell samples using ‘|fold change|=2, *P*<0.05’ as the screening criterion. WGCNA was subsequently used for the screening of genes coexpressed with CHI3L1. The specific steps were as follows: First, a suitable soft threshold was selected to construct a scale-free network that conformed to a power-law distribution to simulate a real biological network more accurately. The blockwiseModules function was subsequently used for module partitioning to define and visualize the gene coexpression modules. By calculating the module eigenvalue (ME), the correlation of each module with the clinical group (tumor group) and CHI3L1 expression was evaluated, and the gene modules with a |correlation coefficient|>0.5 and P<0.05 were screened out. Finally, the DEGs were used for survival analysis via the GSE31312 dataset to identify genes that were significantly associated with the prognosis of lymphoma (*P* < 0.05).

#### 2.3.4 PPI network analysis and functional enrichment analysis

To assess the genes coexpressed with CHI3L1 in lymphoma, we used the STRING database (https://cn.string-db.org/) to perform PPI analysis of the screened genes that were coexpressed with CHI3L1 and obtained a minimum interaction score of 0.7 for the PPI pairs. The PPI network was then constructed via Cytoscape software (version 3.8.0).

To further investigate the biological functions of CHI3L1 in the development and prognosis of lymphoma, we performed functional enrichment analyses of genes in the PPI network that had direct or indirect interactions with CHI3L1. These genes were subjected to Kyoto Encyclopedia of Genes and Genomes (KEGG) pathway and Gene Ontology (GO) functional enrichment analyses via the DAVID database (https://david.ncifcrf.gov). The analyses and statistical tests via Fisher’s exact test revealed significantly enriched KEGG pathways and GO entries (P<0.05), thus revealing the key related biological functions and signaling pathways of CHI3L1 involved in lymphoma.

### 2.4 Expression of the CHI3L1 gene in the PBMCs of patients with aggressive lymphoma measured via RT–qPCR

The PBMC samples from 12 patients with aggressive lymphoma and 10 healthy controls stored in the biobank were thawed, and total RNA was extracted via TRIzol reagent (Invitrogen Life Technologies). Reverse transcription was subsequently performed to obtain cDNA, which was subsequently used for the qPCR assay, and gene primers with the following sequences were designed with Primer 5.0:

CHI3L1 forward primer: 5’-TGGCTTCTTCTGAGACTGGTGTT-3’,
CHI3L1 reverse primer: 5’-CGCTTTCCTGGTCGTAT-3’;
β-actin (H) forward primer: 5’-GTGGCCGAGGACTTTGATTG-3’,
β-actin (H) reverse primer: 5’-CCTGTAACAACGCATCTCATATT-3’.

qPCR was performed according to the manufacturer’s guidelines. Amplification was performed as follows: predenaturation at 95°C for 10 min; 40 PCR cycles (95°C, 10 sec; 60°C, 60 sec). The melting curve program was as follows: 95°C, 10 sec; 60°C, 60 sec; 95°C, 15 sec. All reactions were performed in triplicate and the mRNA levels of the target gene were normalized by β-actin using the 2^-ΔCt^ method.

### 2.5 Statistical analyses

SPSS 26.0 and R 4.0.3 were used for statistical analysis. Measurement information was expressed as the mean ± standard deviation (x ± s), and Mann-Whitney test was used for comparisons between groups; counting information was expressed as the number of cases and percentage [n (%)], and a chi-square test was used for comparisons between groups. Survival analyses were performed via the Kaplan–Meier method with the log-rank test. Spearman correlation analysis was used to assess the correlation between CHI3L1 and clinical indicators in lymphoma patients. A one-way Cox regression model was used to analyze the factors affecting prognosis. *p*<0.05 was considered to indicate a statistically significant difference.

## 3 Results

### 3.1 Analysis of serum CHI3L1 expression and other clinical parameters in invasive lymphoma

**Figure 2:**
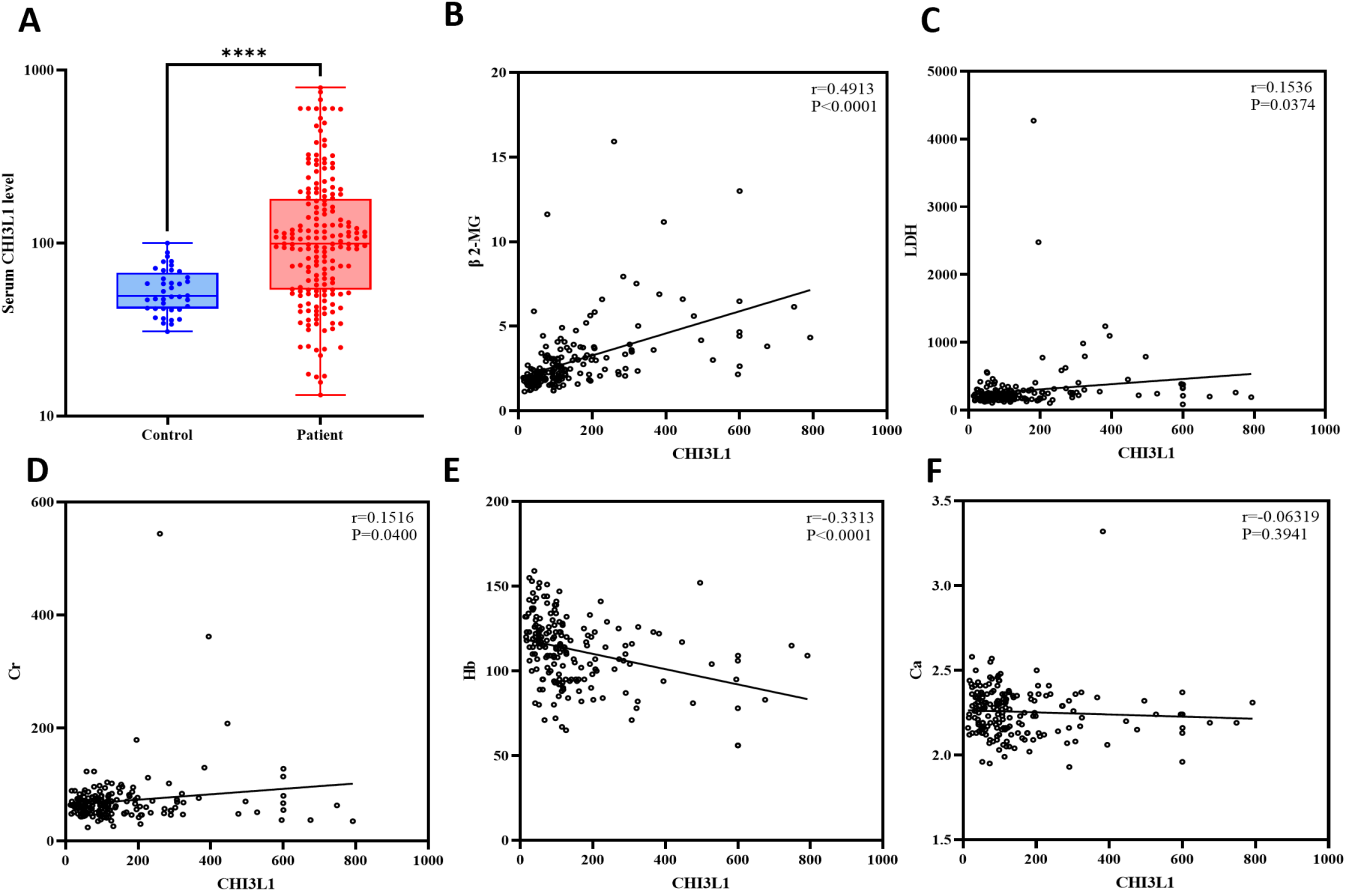
Analysis of the expression levels of the CHI3L1 gene in lymphoma patients and healthy controls and their correlation with clinical indicators. **(A)** Box plot revealing differential expression of CHI3L1 in the serum of lymphoma patients (n=184) and healthy controls (n=40). **(B–F)** Spearman correlation analysis revealed a correlation between CHI3L1 and clinical indicators related to lymphoma. ****P<0.0001.

This study included 184 patients with aggressive lymphoma, encompassing both newly diagnosed and relapsed cases, with 98 male (53.3%) and 86 female (46.7%) patients (**Supplementary Table S1**). We quantified serum CHI3L1 protein expression levels in 184 patients with invasive lymphoma and 40 healthy individuals using ELISA. The results show that serum CHI3L1 levels were significantly elevated in lymphoma patients compared to healthy controls, with a highly significant difference (P < 0.001) (**Figure 2A**). The R package maxstat (version 0.7-25), which applies maximally selected rank statistics with various p-value approximations, was utilized to determine the optimal cutoff value for CHI3L1. The grouping criteria set the minimum sample size to exceed 25% and the maximum sample size to be less than 75%. The final optimal cutoff value was identified as 106.7. Based on this cutoff, the 184 patients with invasive lymphoma were categorized into a CHI3L1 low-expression group (n=127) and a CHI3L1 high-expression group (n=57). We then compared the general clinical characteristics of the two groups. The results showed that in the CHI3L1 high-expression group, a significantly higher proportion of patients were aged ≥60 years, with elevated levels of β2-microglobulin (β2-MG) and lactate dehydrogenase (LDH), as well as reduced hemoglobin (Hb) levels. Additionally, a greater proportion of patients were in advanced stages (III~IV), suggesting that CHI3L1 overexpression may contribute to accelerated disease progression. The International Prognostic Index (IPI) is a widely used scoring system in the field of non-Hodgkin lymphoma, which is employed to evaluate the prognosis of patients. A higher IPI score indicates a poorer prognosis. In this study, the level of CHI3L1 exhibited a significant upward trend with the increase in the IPI score, suggesting a potential correlation between the elevated expression of CHI3L1 and the severity of the disease. The incidence of B symptoms (fever, night sweats, and weight loss) also increased significantly with higher CHI3L1 levels, further reflecting heightened tumor activity (P > 0.05) (**Tables 1**, **Supplementary Tables S2, S3**). Spearman correlation analysis revealed that serum CHI3L1 levels in invasive lymphoma patients were positively correlated with β2-MG, LDH, and creatinine (Cr), but negatively correlated with Hb. No significant correlations were found between CHI3L1 levels and serum calcium (Ca)、CRP levels (P > 0.05) (**Figures 2B–F**, **Supplementary Figure S4**).

**Table 1:** Comparison of the general data of patients with aggressive lymphoma in the CHI3L1 high-expression group and low-expression group.

Index	CHI3L1 low expression group (<106.7) (n=127)	CHI3L1 high expression group (≥106.7) (n=57)	x value	p value
Gender, n (%)			0.188	0.664
male	69 (37.5)	29 (15.8)		
female	58 (31.5)	28 (15.2)		
Age, n (%)			8.736	0.003
≥60 years old	57 (31.0)	39 (21.2)		
<60 years old	70 (38.0)	18 (9.8)		
β-MG, n (%)			42.610	0.000
≥2.62	31 (16.8)	43 (23.4)		
<2.62	96 (52.2)	14 (7.6)		
LDH, n (%)			19.447	0.000
≥248	31 (16.8)	33 (17.9)		
<248	96 (52.2)	24 (13.0)		
Hb, (g/L)			13.254	0.000
≥106	95 (51.6)	27 (14.7)		
<106	32 (17.4)	30 (16.3)		
PLT, (10^9/L)			0.015	0.902
≥199	48 (26.1)	21 (11.4)		
<199	79 (42.9)	36 (19.6)		
TCa, (mmol/L)			4.626	0.031
≥2.25	73 (39.7)	23 (12.5)		
<2.25	54 (29.3)	34 (18.5)		
Cr, (umol/L)			5.313	0.021
≥72	32 (17.4)	24 (13.0)		
<72	95 (51.6)	33 (17.9)		
Ann Arbor staging, n (%)			11.562	0.001
I-II	70 (38.0)	16 (8.7)		
III–IV	57 (31.0)	41 (22.3)		
symptom, n (%)			5.829	0.016
with group B symptoms	45 (24.5)	31 (16.8)		
without group B symptoms	82 (44.6)	26 (14.1)		
IPI score, n (%)			13.990	0.000
≥2	33 (17.9)	31 (16.8)		
<2	94 (51.1)	26 (14.1)		

### 3.2 Serum CHI3L1 levels and other clinical indicators in lymphoma patients: association with disease staging

**Figure 3:**
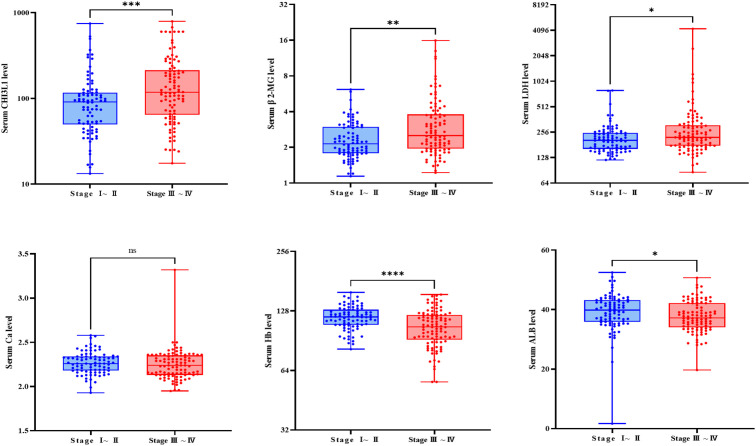
Comparison of relevant clinical examination parameters between patients with different stages. Box plot showing the differential expression of CHI3L1, β 2-MG, LDH, Ca, and ALB in the serum of patients with stage III-IV lymphoma (n=98) and patients with stage I-II lymphoma (n=86). ns indicates non-significant difference (P > 0.05), *P<0.05, **P<0.01, ***P<0.001, ****P<0.0001.

Analysis of clinical indicators based on Ann Arbor staging revealed that CHI3L1 levels were significantly higher in stage III-IV patients than in stage I-II patients (P = 0.001), suggesting that this biomarker is associated with tumor progression. An analysis of other clinical characteristics showed that stage III-IV patients had markedly abnormal levels of albumin, hemoglobin (Hb), β2-microglobulin (BMG), and lactate dehydrogenase (LDH), indicating malnutrition, severe anemia, and an increased tumor burden in advanced-stage patients. Conversely, no significant differences were observed between stages in terms of age, fibrinogen, CRP, creatinine, serum calcium, or white blood cell count (P > 0.05) (**Figure 3**, **Supplementary Figures S4, S5**).

### 3.3 Elevated CHI3L1 levels are associated with poor prognosis and reduced OS in aggressive lymphoma

**Figure 4:**
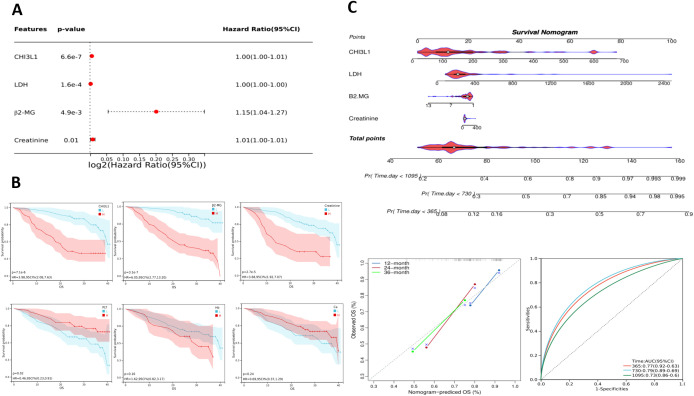
Prognostic analysis of lymphoma patients. **(A)** Single-factor Cox analysis forest plot of laboratory test parameters that may affect the prognosis of lymphoma patients. **(B)** Estimated OS among patients grouped by CHI3L1, β2-MG, Cr, PLT, Hb, and Ca levels according to Kaplan–Meier analysis. **(C)** Nomogram for predicting the OS of invasive lymphoma patients based on risk factors screened through univariate analysis; calibration curves and ROC curves of the nomogram.

To reduce the influence of treatment interventions and variations in disease progression on the follow-up study results, this study independently analyzed 92 newly diagnosed patients selected from a total of 184. This selection aimed to provide a more precise assessment of the association between CHI3L1 expression levels and disease progression. A univariate Cox proportional hazards regression model was employed to examine the relationship between clinical characteristics, laboratory parameters, and patient prognosis (**Supplementary Table S6**). Elevated levels of CHI3L1, LDH, β_2_-MG, and Cr were significantly associated with shorter OS (all P < 0.05), whereas sex, age, Ca, Hb, and PLT were not significantly associated with prognosis (all P > 0.05) (**Figure 4A**, **Supplementary Table S7**). Patients were categorized into high and low CHI3L1 expression groups based on an optimal cutoff value. Kaplan–Meier survival curves were constructed to evaluate prognostic differences using the log-rank test. The analysis demonstrated a statistically significant difference in OS between the high-expression and low-expression CHI3L1 groups (P < 0.001), with higher CHI3L1 levels associated with shorter OS. These findings indicate that CHI3L1 may serve as a potential prognostic biomarker to inform individualized treatment strategies. In addition, OS was significantly shorter in patients with elevated β_2_-MG levels, elevated Cr levels, and low PLT counts (**Figure 4B**). Nomograms with scores of 0-100 can be developed according to the Cox proportional risk model correlation coefficients of each variable; the sum of the scores of each variable represents the total score, and the overall survival rate of a specific patient can be derived from the downward projection of the scale of the total score at 18 months, 36 months and 60 months. In this study, a nomogram prediction model for OS in aggressive lymphoma patients was constructed by integrating four indicators, namely, CHI3L1, LDH, β2-MG, and Cr. The performance of the model was then comprehensively evaluated in terms of the differentiation index (including the C index, time-ROC, and time-AUC) and calibration, which provided a reference for clinical diagnosis and treatment (**Figure 4C**).

### 3.4 Elevated CHI3L1 expression: a diagnostic and prognostic biomarker in lymphoma

**Figure 5:**
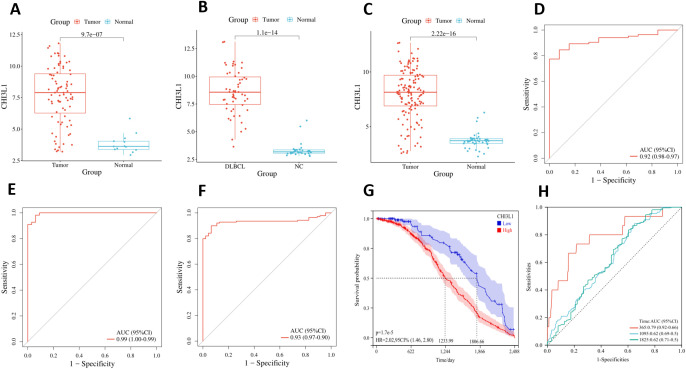
Differential expression, diagnostic value, and prognostic significance of CHI3L1 in lymphoma. Differential expression of CHI3L1 in the normal and tumor groups was analyzed on the basis of **(A)** the GSE25638 dataset, **(B)** the GSE56315 dataset, and **(C)** the merged dataset. ROC curve analysis revealed the diagnostic value of CHI3L1 in lymphoma on the basis of **(D)** the GSE25638 dataset, **(E)** the GSE56315 dataset, and **(F)** the merged dataset. **(G)** Kaplan–Meier survival curves based on the GSE31312 dataset were generated to analyze the relationship between CHI3L1 expression and lymphoma prognosis. **(H)** ROC curves based on the GSE31312 dataset were used to analyze the ability of CHI3L1 to predict the survival outcomes of patients with lymphoma at different time points.

Using the GSE25638, GSE56315, and merged datasets, we analyzed CHI3L1 expression differences between lymphoma samples (tumor group) and normal control samples (normal group). Across all datasets, CHI3L1 was significantly upregulated in the tumor group (P < 0.05; **Figures 5A–C**). ROC curve analysis demonstrated high diagnostic accuracy, with area under the curve (AUC) values of 0.92, 0.99, and 0.93 for the respective datasets (**Figures 5D–F**). Analysis of the GSE31312 dataset revealed that high CHI3L1 expression was strongly associated with poor lymphoma prognosis, as shown by Kaplan–Meier survival analysis (P < 0.05; **Figure 5G**). Prognostic ROC analysis further demonstrated AUCs of 0.79, 0.62, and 0.62 for predicting 1-, 3-, and 5-year survival, respectively (**Figure 5H**). These findings indicate that CHI3L1 is overexpressed in lymphoma cells and has potential diagnostic and prognostic value.

### 3.5 Screening of genes associated with CHI3L1 in lymphomas

**Figure 6:**
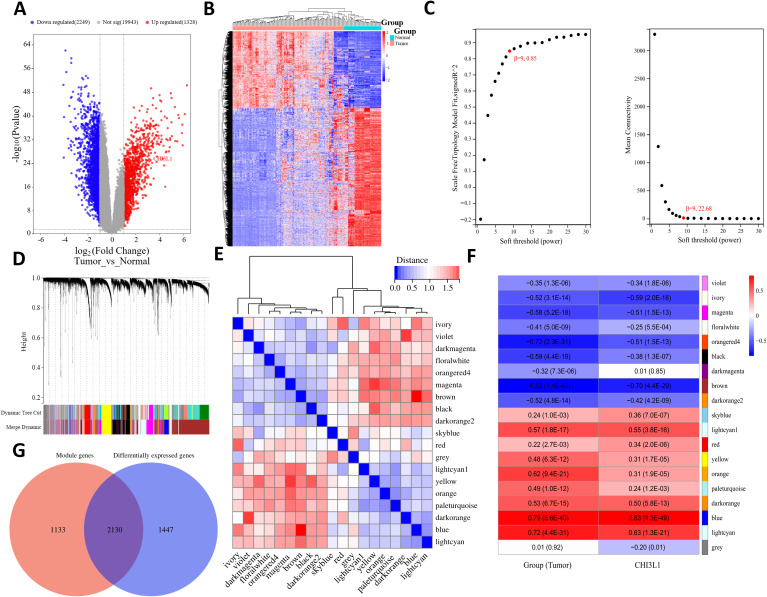
Differential expression analysis and WGCNA revealed genes associated with CHI3L1 expression in lymphoma. **(A)** Volcano plot showing the differentially expressed genes in the normal and tumor groups. **(B)** Heatmap showing the expression patterns of the DEGs in each sample. **(C)** Plot of the different soft thresholds (power values, x-axis) versus the network connectivity fitting index R ^ 2 (y-axis) and the average connectivity (average node degree) (y-axis). The best soft threshold of β 9 was chosen for this study. **(D)** Systematic clustering tree of genes divided by gene expression pattern similarity on the basis of the best soft threshold β. Different colors represent the identified gene modules. **(E)** Cluster plot of module eigengenes (MEs) and heatmap of connectivity between modules. The color shade of each square represents the magnitude of the corresponding intermodule connectivity. **(F)** Heatmap of the correlation between the feature value (ME) of gene modules and the tumor group and CHI3L1 expression, where the module name is represented in different colors, and the value within each square represents the correlation coefficient and P value of the module feature vector (ME) and phenotype. **(G)** Venn diagram of the CHI3L1-associated gene sets.

A total of 3577 genes that were differentially expressed between the tumor and normal groups were screened from the combined GSE25638 and GSE56315 datasets (|fold change| > 2, P < 0.05), including 1328 genes that were significantly upregulated and 2249 genes that were significantly downregulated in the tumor group (**Figures 6A, B**). To ensure that the network was scale-free and more biologically meaningful, the optimal soft threshold β was set to 9 (**Figure 6C**). Then, on the basis of the dynamic tree cut algorithm, the minimum module gene number was set to 30, the sensitivity dynamic tree cutting (depth segmentation) was set to 3, and the maximum module distance was set to 0.25 to generate the gene modules and further merge the modules with high similarity. As shown in **Figure 6D**, 19 gene modules were finally generated after merging: violet, ivory, magenta, floralwhite, orangered4, black, darkmagenta, brown, darkorange2, skyblue, lightcyan1, red, yellow, orange, pale turquoise, darkorange, blue, lightcyan, and gray. We analyzed the connectivity of the module eigengenes (MEs), and the results revealed that the distance between the modules was greater than 0.25 (**Figure 6E**), which indicated good independence between each module. The correlation between each gene module and clinical subgroup as well as CHI3L1 expression was subsequently calculated, and a total of 9 gene modules were determined to be closely correlated with both the clinical subgroup and CHI3L1 expression (|correlation coefficient|>0.5, P<0.05); these modules were the brown, blue, lightcyan, lightcyan1, magenta, ivory, darkorange, and orangered4 modules and contained a total of 3263 genes (**Figure 6F**). A total of 2130 common genes were obtained from the comparison of these module genes and DEGs (**Figure 6G**).

### 3.6 PPI network analysis and functional enrichment analysis

**Figure 7:**
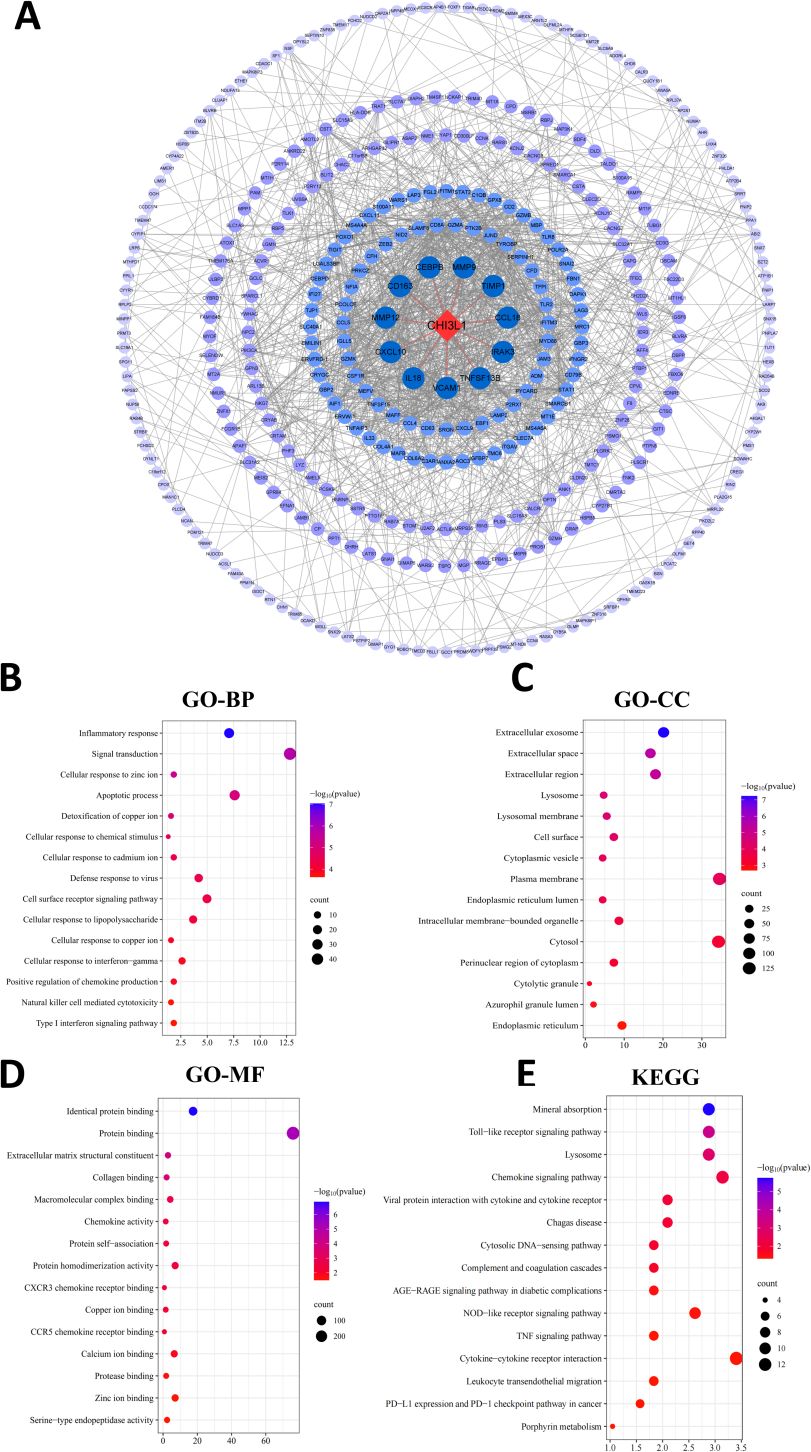
PPI network analysis and functional enrichment analysis. **(A)** PPI network constructed via the STRING database. The blue circles indicate proteins that interact with CHI3L1. A darker blue color indicates stronger interaction ability, and proteins that directly interact with CHI3L1 are connected with a line. **(B–D)** GO functional enrichment analyses showing the top 15 most significant terms in the categories of **(B)** biological process, **(C)** cellular component, and **(D)** molecular function. **(E)** KEGG pathway enrichment analysis (top 15 entries with the highest significance).

PPI analysis of genes associated with CHI3L1 expression in lymphoma was performed, and the results are shown in **Figure 7A**. In the PPI network, 385 nodes were included, of which 11 nodes had direct interactions with CHI3L1 and 373 nodes had indirect interactions with CHI3L1, indicating that CHI3L1 is involved in the development and prognosis of lymphomas through interactions with these genes. To investigate the biological functions of CHI3L1 in lymphoma development and prognosis in detail, we further performed functional enrichment analysis of the genes in the PPI network that have potential regulatory roles with CHI3L1. The results of the GO analysis revealed that these genes are involved in 43 biological processes (BPs), such as the inflammatory response, signal transduction, the cellular response to zinc ions, the apoptotic process, and the detoxification of copper ions (**Figure 7B**), and are involved in 41 cellular components (CCs), such as the extracellular exosome, extracellular space, extracellular region, lysosomal membrane, and lysosome (**Figure 7C**). At the molecular function level, these genes are related to 21 main molecular functions (MFs), such as protein binding, extracellular matrix structural constituent, collagen binding, and macromolecular complex binding (**Figure 7D**). KEGG enrichment analysis revealed that genes directly or indirectly interacting with CHI3L1 in these lymphomas were enriched mainly in mineral absorption, the Toll-like receptor signaling pathway, lysosomes, the chemokine signaling pathway, the NOD-like receptor signaling pathway and 20 other signaling pathways (**Figure 7E**).

### 3.7 The level of CHI3L1 gene expression in the PBMCs of lymphoma patients is greater than that in the PBMCs of healthy individuals

**Figure 8:**
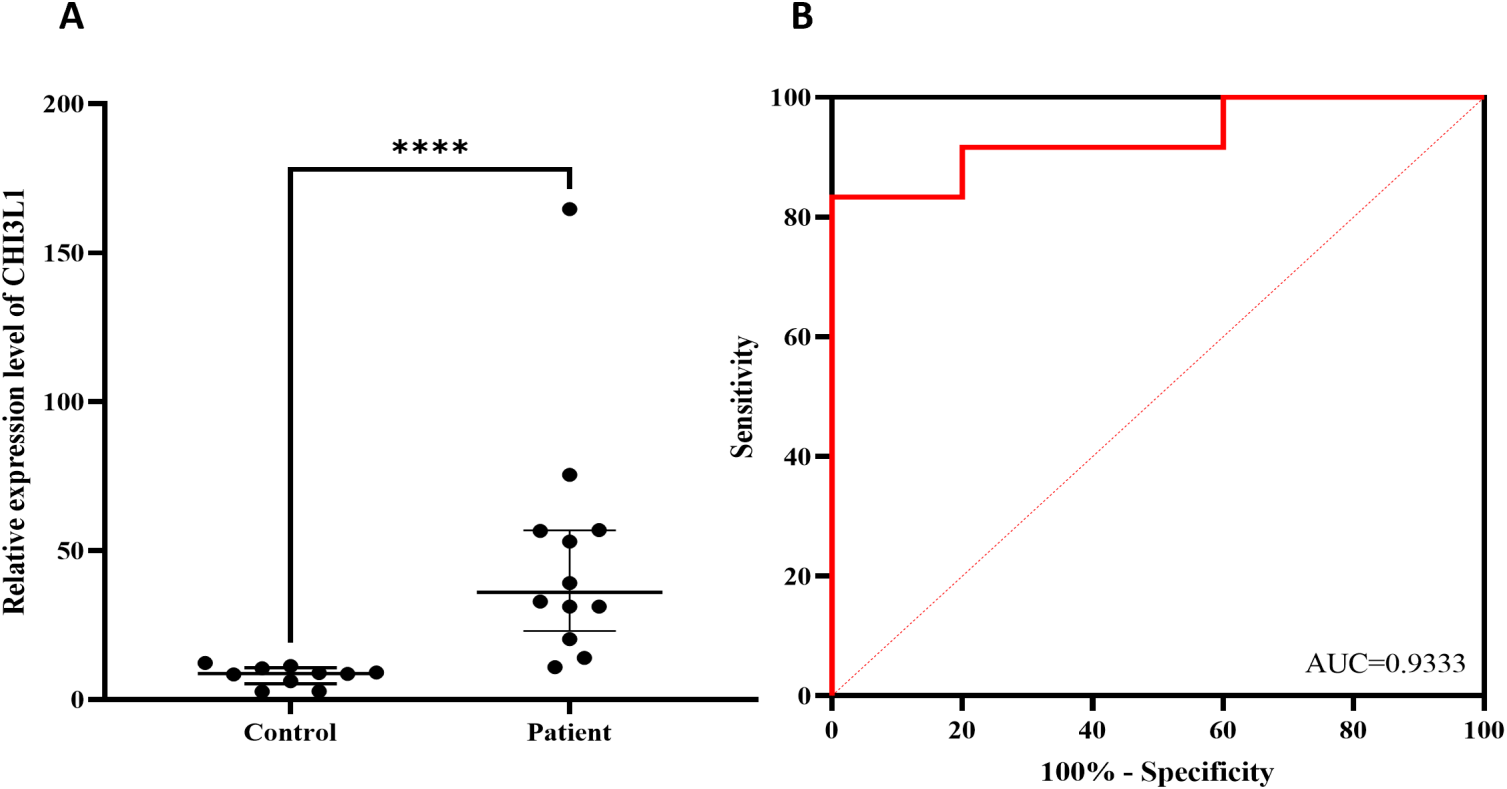
Verify the differential expression of CHI3L1 using RT-qPCR. **(A)** CHI3L1 mRNA expression levels in lymphoma patients (n=12) and healthy controls (n=10) and **(B)** ROC curves according to the RT–qPCR results. ****P<0.0001.

The expression of the CHI3L1 gene in PBMCs from 12 patients with aggressive lymphoma and 10 healthy individuals was detected via RT–qPCR. The results revealed that the expression level of the CHI3L1 gene in patients with aggressive lymphoma was significantly greater than that in healthy controls (P < 0.01) (**Figure 8A**). ROC analysis revealed that the AUC of CHI3L1 for predicting aggressive lymphoma was 0.933 (95% CI: 0.825~1.000), with a sensitivity of 83.3% and a specificity of 100% (**Figures 8B**, **Supplementary Figure S8**).

## Discussion

In this study, we systematically combined bioinformatics analysis and experimental validation to thoroughly investigate the expression level of CHI3L1 in aggressive lymphomas and its prognostic value. Our results indicate that CHI3L1 is highly expressed in aggressive lymphomas and is closely associated with poor prognosis.

CHI3L1, a glycoprotein, has multiple biological functions in the tumor microenvironment. CHI3L1 is involved in inflammation, cell proliferation and differentiation, apoptosis, angiogenesis promotion and extracellular matrix remodeling (6, 20). CHI3L1 can directly stimulate tumor cells to produce proinflammatory cytokines (IL-6, IL-8, and transforming growth factor-β) and activate the transforming growth factor-β signaling pathway through binding to its receptor, resulting in proinflammatory cytokine production and immune cell recruitment. The production of proinflammatory cytokines (IL-6, IL-8 and TGF-β) activates the TGF-β signaling pathway by binding to its receptor, resulting in the production of proinflammatory cytokines and the recruitment of immune cells, which promotes tumor cell migration and invasion and indirectly participates in tumor progression by promoting angiogenesis in the tumor microenvironment (8, 21, 22). In cutaneous T-cell lymphomas (CTCLs), Hideko Suzuki et al. (23) reported that serum levels of CHI3L1 were elevated and positively correlated with the severity of the disease. positively correlated with disease severity. Immunohistochemical analysis revealed that CHI3L1 was expressed in both epidermal keratinocytes and tumor cells in CTCL lesions. Although CHI3L1 did not significantly affect cytokine production in the CTCL cell line, it promoted the proliferation of Hut 78 and HH cells *in vitro* by activating the ERK1/2 pathway. It was hypothesized that CHI3L1 may activate the ERK1/2 pathway through autocrine and paracrine effects, thereby driving the proliferation of CTCL cells and promoting disease progression. Other studies have shown that CHI3L1 can stimulate the phosphorylation of ERK1/2 in human renal epithelial cell lines (293 cells) and human brain astrocytoma cells (U373 and U87 MG cells), which leads to cell proliferation (24).

By analyzing the GSE25638 and GSE56315 datasets, we found that CHI3L1 expression in lymphoma samples was significantly higher than that in normal samples, suggesting that high CHI3L1 expression may be closely related to the occurrence and progression of lymphomas. ROC curve analysis revealed that CHI3L1 has high sensitivity and specificity in the diagnosis of lymphomas, suggesting that it is a potential diagnostic biomarker. For experimental validation, we used qPCR to detect CHI3L1 gene expression in peripheral blood single nucleated cells, and the results revealed that the expression of CHI3L1 in patients with aggressive lymphoma was significantly greater than that in healthy controls. ELISA analysis of serum samples revealed that the protein level of CHI3L1 in patients with lymphoma was significantly greater than that in healthy controls, that the serum CHI3L1 protein level in patients with stage III-IV lymphoma was significantly greater than that in healthy controls, and that the serum CHI3L1 protein level in patients with stage III-IV lymphoma was significantly greater than that in patients with stage I-II lymphoma. Patients with significantly higher serum CHI3L1 protein levels. These results further support the association between CHI3L1 and disease progression. More importantly, follow-up of 184 patients with aggressive lymphoma revealed that patients in the nonsurviving group had significantly higher CHI3L1 levels than did those in the surviving group. These findings suggest that high CHI3L1 expression may predict faster disease progression or worse prognosis in lymphoma patients. For patients with high CHI3L1 expression, more aggressive treatments, such as combination immunotherapy, molecular targeted therapy, or novel chemotherapeutic regimens, may be needed; moreover, monitoring the expression levels of CHI3L1 and related genes can be used to assess treatment efficacy and disease progression and guide clinical decision-making.

CHI3L1, an indicator of the inflammatory and immune status of the body, has been shown to be associated with the prognosis of patients with malignant tumors such as colon, breast, ovarian, and non-small cell lung cancers (25–29). Qiu et al. (14)demonstrated that CHI3L1 plays a particularly prominent role in hepatocellular carcinoma, where it promotes the growth and metastasis of liver tumors by regulating SMAD family members (SMAD-2 and SMAD-3), affecting the TGF-β signaling pathway and thereby promoting liver tumor growth and metastasis. Based on this mechanism, targeting CHI3L1 may be a potential therapeutic strategy to inhibit hepatocellular carcinoma growth and metastasis. Yu et al. identified significantly elevated CHI3L1 levels in the serum and tissues of lung cancer patients compared to the control group. Their study developed a humanized anti-CHI3L1 antibody, which exhibited strong anti-tumor and anti-metastatic effects in a lung cancer mouse model, effectively suppressing tumor growth and dissemination. These effects were closely associated with the inhibition of STAT6-dependent M2 macrophage polarization. Furthermore, proteomic analysis revealed that plasminogen (PLG) interacts with CHI3L1, playing a crucial role in regulating the M2 polarization process (30). Chiang et al. (31), on the other hand, examined the expression of CHI3L1 in 180 patients with epithelial ovarian carcinomas via RT–qPCR and reported that late-stage and chemotherapy-resistant patients presented significantly increased CHI3L1 expression and that patients with high CHI3L1 expression presented decreased progression-free survival (PFS) and OS. C-reactive protein (CRP), a well-established inflammatory marker, is widely used to assess inflammation and immune response across various diseases. However, this study found no significant correlation between CHI3L1 and CRP levels. Although both are involved in immune and inflammatory processes, their mechanisms of action differ. CHI3L1 primarily regulates cytokines, mediates cellular injury responses, and contributes to fibrosis, whereas CRP, an acute-phase reactant, is predominantly synthesized in the liver and induced by inflammatory mediators such as IL-6. The expression and regulation of these markers are likely influenced by distinct physiological and pathological mechanisms, which may explain the lack of a direct correlation.

In our study, through prognostic analysis of the GSE31312 dataset, we found that high expression of CHI3L1 was strongly associated with poor prognosis in patients with aggressive lymphomas. K–M survival analysis further confirmed that the survival rate of patients with high CHI3L1 expression was significantly lower than that of patients with low CHI3L1 expression. These findings suggest that CHI3L1 not only plays an important role in the diagnosis of lymphoma but is also a potential prognostic marker. In conclusion, Cox regression analysis revealed that CHI3L1, LDH, β2-MG and Cr were all independent factors affecting the prognosis of aggressive lymphoma patients. This finding reinforces the importance of CHI3L1 in prognostic assessment. As these factors independently predict patient prognosis, clinicians can develop personalized treatment plans based on these biomarkers. In particular, after these prognostic factors were integrated, we constructed a column-line graph prediction model for OS in patients with aggressive lymphoma. The model provides clinicians with an intuitive and practical tool to help them predict patient survival more accurately. For internal validation, the C-index (C-index) of the column-line diagram exceeded 0.700 in both the training and validation sets, indicating the high accuracy of the model in distinguishing patient prognosis. In addition, ROC curve analysis revealed that the AUC values of the model for predicting patient OS at 12, 24 and 36 months were 0.77, 0.79 and 0.73, respectively, indicating its stable and reliable predictive ability. The calibration curves further indicated that the model had good fitting ability, supporting its feasibility in clinical applications. With the accumulation of more clinical data, the column-line graph prediction model is expected to be further optimized and become an important tool for assessing the individualized risk of patients with aggressive lymphoma.

To elucidate the molecular mechanisms underlying CHI3L1 in aggressive lymphoma, this study analyzed the GSE25638 and GSE56315 datasets, identifying nine gene modules strongly associated with both clinical classification and CHI3L1 expression. Univariate Cox regression analysis revealed that 605 genes were significantly correlated with lymphoma prognosis, reinforcing the potential of CHI3L1 as a prognostic biomarker.Protein-protein interaction (PPI) network analysis identified 11 genes that directly interact with CHI3L1 and 373 genes that interact indirectly. These findings suggest that CHI3L1 may facilitate tumor cell growth and dissemination by regulating critical biological processes, including cell proliferation, apoptosis, and migration. Furthermore, the identified interactions indicate that CHI3L1 may contribute to lymphoma progression through multiple signaling pathways and molecular networks.We performed Gene Ontology (GO) and Kyoto Encyclopedia of Genes and Genomes (KEGG) enrichment analyses on the protein-protein interaction (PPI) network of CHI3L1-related genes. The GO analysis revealed that these genes are primarily localized in extracellular vesicles, the extracellular matrix, and lysosomal membranes. They are closely associated with protein binding, extracellular matrix components, and collagen binding. Functionally, they are involved in biological processes such as the inflammatory response, signal transduction, apoptosis, and cellular responses to metal ions and detoxification. These findings suggest that CHI3L1-related genes play a regulatory role in tumor cell proliferation, invasion, and survival. The KEGG pathway enrichment analysis indicated that these genes are significantly enriched in mineral absorption, the Toll-like receptor signaling pathway, and the chemokine signaling pathway. These pathways may contribute to tumor metastasis by modulating immune and inflammatory responses.TLR4 (Toll-like receptor 4) is a critical member of the Toll-like receptor family and plays a fundamental role in the innate immune system. Studies have demonstrated that stimulation with the TLR4 ligand LPS downregulates CHI3L1 expression in SW480 colorectal cancer cells. The D299G and T399I mutations in TLR4 result in a loss of function, thereby inhibiting TLR4 signaling. Further research has shown that in CaCO2 cells harboring these mutations, CHI3L1 mRNA levels are significantly elevated, establishing a direct correlation between TLR4 mutations and CHI3L1 expression.Evidence suggests that TLR4 mutations and CHI3L1 upregulation may synergistically promote carcinogenic alterations in colonic epithelial cells within an inflammatory environment. Specifically, the D299G mutation disrupts cellular inflammatory response mechanisms, potentially activating the Wnt or PI3K/AKT pathway. This activation indirectly enhances CHI3L1 expression, contributing to tumor progression. Moreover, the D299G mutation is associated with higher tumor grades and increased metastasis rates, underscoring the pivotal role of TLR4 mutations and CHI3L1 upregulation in colorectal cancer development and progression (32). Previous studies have demonstrated that CHI3L1 expression is significantly upregulated in the lungs of mice bearing breast tumors. *In vitro* experiments further revealed that recombinant CHI3L1 stimulates pulmonary interstitial and alveolar macrophages to secrete CCL2, CXCL2, and MMP9. The elevated levels of these factors facilitate the metastasis of breast tumor cells to the lungs (33). Wang et al. demonstrated that CHI3L1 enhances the proliferation of CD4^+^ T cells, CD8^+^ T cells, and B cells in PTLCs. The underlying mechanism of CHI3L1-induced lymphocyte proliferation involves multiple signaling pathways, including NF-κB, MAPK (c-Jun N-terminal kinase [JNK] and ERK), TGF-β1/Smad, and Akt (34).Based on the above analysis, CHI3L1 may affect the development and progression of aggressive lymphoma by regulating key biological processes, such as the inflammatory response, apoptosis, signal transduction, and extracellular matrix remodeling; its association with multiple key signaling pathways suggests that CHI3L1 has potential as a therapeutic target. Targeted intervention with CHI3L1 may inhibit the proliferation and invasion of tumor cells, thereby improving patient prognosis.

In this study, we systematically investigated the expression characteristics of CHI3L1 in aggressive lymphoma and its significance in disease diagnosis and prognosis. These results indicate that CHI3L1 is involved in the development and progression of aggressive lymphoma, suggesting that it may be a promising biomarker and therapeutic target, which could help to elucidate the molecular mechanisms of aggressive lymphoma and promote the development of novel diagnostic tools and therapeutic strategies. It is important to highlight that while our study identified a significant increase in CHI3L1 expression in lymphoma cells, existing literature indicates that other cell types within the tumor microenvironment such as macrophages, fibroblasts, and neutrophils may also play pivotal roles in regulating CHI3L1 expression (35). These cells contribute to CHI3L1 secretion and are actively involved in critical biological processes, including inflammatory responses, apoptosis, and signal transduction, all of which can profoundly influence tumor progression and prognosis. Although our study primarily focuses on CHI3L1 expression in lymphoma cells, the tumor microenvironment is a highly intricate network of cellular interactions. Thus, the contribution of other cellular components to CHI3L1 secretion should not be overlooked. To gain a more comprehensive understanding of this mechanism, future research will utilize immunohistochemistry (IHC) and *in situ* hybridization (ISH) to examine CHI3L1 expression across various cell types within tumor tissue sections. These approaches will provide deeper insights into the specific roles of CHI3L1 in both tumor cells and the surrounding microenvironment. Additionally, sample size limitations may affect the generalizability and credibility of the results, and smaller sample sizes may not be able to encompass all potential biological variants, thus limiting the extrapolation of the study findings. Future studies should expand the sample size, incorporate multicenter data for validation, and focus on the specific mechanism of action of CHI3L1 in aggressive lymphomas, especially its interactions with key signaling pathways and cellular processes. Moreover, larger-scale clinical studies should be conducted to verify the reliability and validity of CHI3L1 as a diagnostic and prognostic marker and to explore its feasibility in clinical practice. Through in-depth molecular mechanism studies and clinical validation, CHI3L1 is expected to be a key target in the diagnosis and treatment of aggressive lymphomas, and the survival rate and quality of life of patients will improve.

## Data Availability

The original contributions presented in the study are included in the article/**Supplementary Material**, further inquiries can be directed to the corresponding author/s.
