# Supplementary material for: Abnormally high expression of CHI3L1 in peripheral blood mononuclear cells and serum and their potential diagnosis and prediction from lymphoma patients
Source: Front Immunol. 2025 Apr 7;16:1557802. doi: 10.3389/fimmu.2025.1557802 (PMC12009859; doi:10.3389/fimmu.2025.1557802)
Supplement: Supplementary file 1 [file Table1.docx]

**S1. The characteristics of the studied patients**

| Clinical characteristics | Number of patients (cases) | Percentage (%) |
| --- | --- | --- |
| Sex |  |  |
| Male | 98 | 53.3 |
| Female | 86 | 46.7 |
| Age |  |  |
| >60 years old | 92 | 50 |
| ≤60 years old | 92 | 50 |
| B symptoms |  |  |
| With B symptoms | 76 | 41.3 |
| No B symptoms | 108 | 58.7 |
| Clinical Stage |  |  |
| Stage I | 16 | 8.7 |
| Stage II | 70 | 38.0 |
| Stage III | 39 | 21.2 |
| Stage IV | 59 | 32.1 |
| IPI score |  |  |
| 0~1 points | 68 | 37 |
| 2~3 points | 83 | 45.1 |
| 4~5 points | 33 | 17.9 |
|  |  |  |

**S2. Comparison of Clinical Characteristics and Laboratory Parameters in Patients With and Without B Symptoms**

| Subgroups | With B symptoms | Without B symptoms | U-value | *P-value* |
| --- | --- | --- | --- | --- |
| Number of cases | 76 | 108 |  |  |
| Age(year) | 61.0（55.0-68.0） | 60.0（55.0-67.0） | 3968.5 | 0.703 |
| FIB(g/L) | 3.26（2.67-4.04） | 3.00（2.50-3.84） | 3525.5 | 0.137 |
| Albumin(g/L) | 36.6（33.9-41.5） | 39.1（35.8-43.0） | 3168.0 | 0.009 |
| Creatinine(μmol/L) | 65.5（51.0-86.8） | 63.0（50.0-72.8） | 3572.5 | 0.135 |
| Calcium(mmol/L) | 2.24（2.16-2.33） | 2.27（2.15-2.36） | 3887.5 | 0.543 |
| Hb(g/L) | 109.0（95.0-119.8） | 118.0（101.3-128.8） | 3163.5 | 0.008 |
| WBC(10^9/L) | 4.65（3.50-6.30） | 4.78（3.54-6.25） | 4088.0 | 0.964 |
| PLT(10^9/L) | 158.5（121.0-241.5） | 174.0（128.5-226.8） | 3852.5 | 0.480 |
| β2-MG(mg/L) | 2.83（2.02-3.90）） | 2.11（1.81-2.95） | 2878.5 | 0.001 |
| LDH(U/L) | 223.0（171.0-332.8） | 207.0（169.0-254.0） | 3486.5 | 0.083 |
| CHI3L1 (ng/mL) | 107.81（73.02-206.87） | 97.82（50.20-135.46） | 3265.0 | 0.018 |

**S3. Comparison of Clinical and Biochemical Parameters Among Patients in Different IPI Score Groups**

| Grouping | 0~1 point | 2~3 point | 4~5 point | H-Value | *P-*Value |
| --- | --- | --- | --- | --- | --- |
| Number of cases | 68 | 83 | 33 |  |  |
| Age | 57.0（51.0-61.8） | 63.0（55.0-68.0） | 67.0（62.0-70.0） | 23.047 | 0.000 |
| FIB(g/L) | 2.82（2.49-3.28） | 3.24（2.59-3.89） | 3.88（2.95-4.70） | 14.578 | 0.001 |
| Albumin(g/L) | 40.5（36.2-43.5） | 37.7（34.6-42.3） | 36.8（34.5-40.4） | 7.788 | 0.020 |
| Creatinine(μmol/L) | 64.5（52.3-73.0） | 63.0（49.0-78.0） | 65.0（50.5-80.5） | 0.116 | 0.943 |
| Calcium(mmol/L) | 2.27（2.19-2.34） | 2.23（2.13-2.35） | 2.29（2.16-2.36） | 2.576 | 0.276 |
| Hb(g/L) | 119.5（109.0-132.8） | 111.0（94.0-123.0） | 108.0（95.0-119.5） | 11.463 | 0.003 |
| WBC(10^9/L) | 4.78（3.54-6.25） | 4.88（3.54-6.81） | 4.18（3.14-5.40） | 1.406 | 0.495 |
| PLT(10^9/L) | 166.5（122.0-219.5） | 180.0（121.0-262.0） | 153.0（127.5-210.0） | 1.025 | 0.599 |
| β2-MG(mg/L) | 1.94（1.72-2.47） | 2.33（1.93-3.60） | 3.15（2.36-4.38） | 29.700 | 0.000 |
| LDH(U/L) | 176.5（152.3-221.5） | 232.0（184.0-301.0） | 263.0（188.5-325.0） | 27.088 | 0.000 |
| CHI3L1 (ng/mL) | 76.75（42.09-115.35） | 107.35（60.74-201.33） | 161.67（91.41-280.18） | 21.290 | 0.000 |

**S4.The correlation between serum CRP concentration and CHI3L1 concentration in patients with lymphoma, and the differential expression of CRP concentration between patients with different stages of lymphoma.**


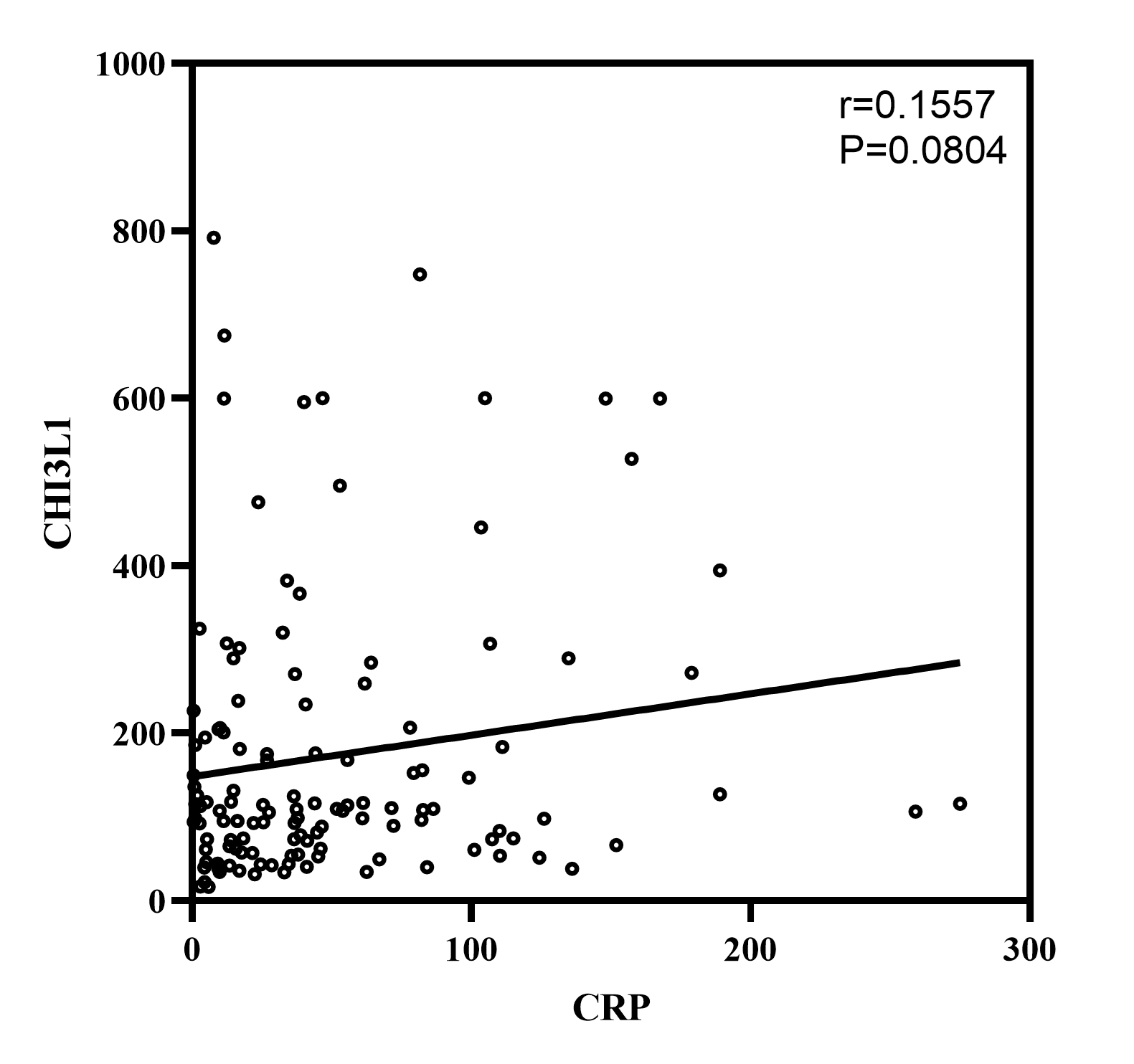

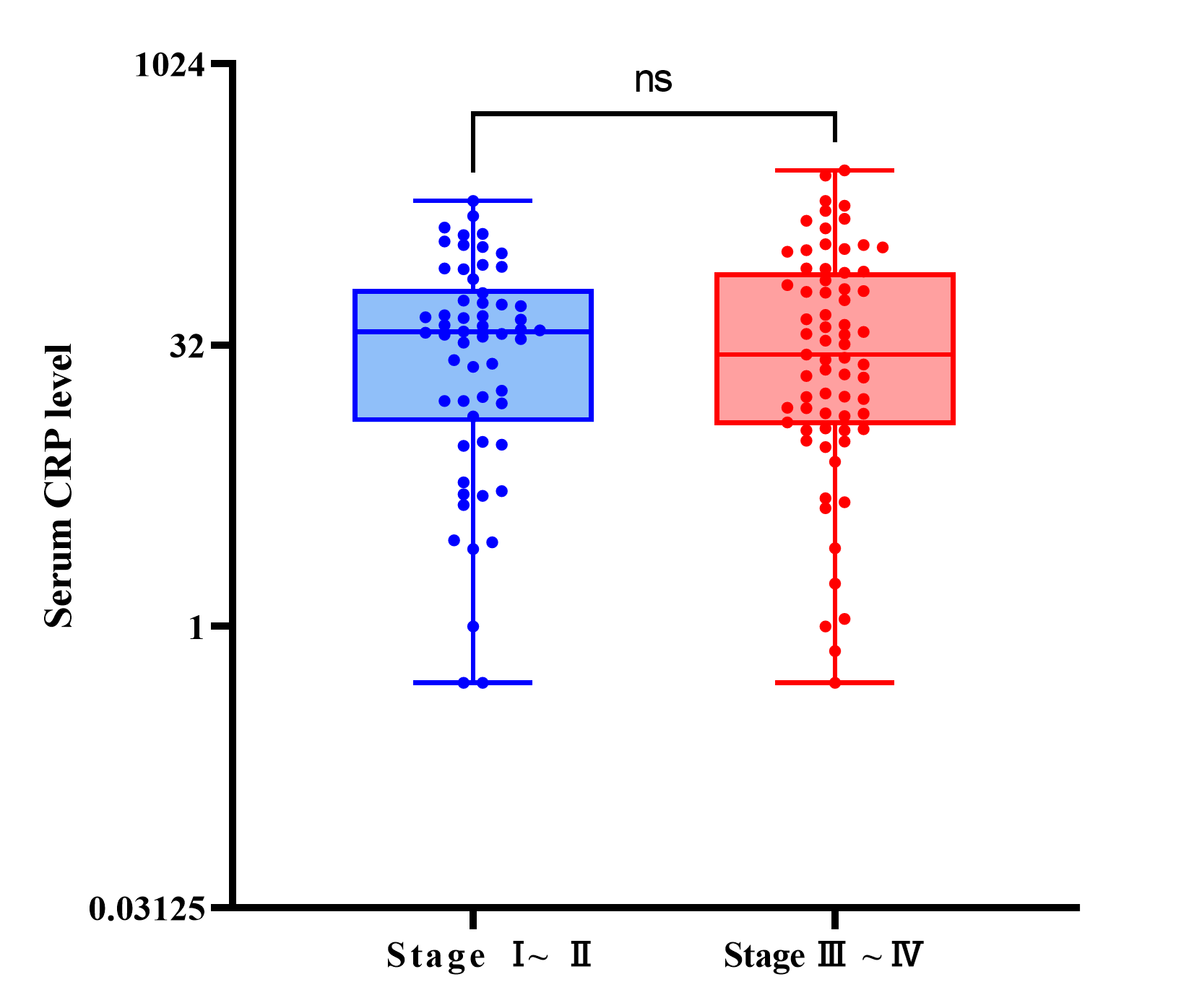


**S5. Comparison of Clinical Characteristics and Laboratory Parameters Across Different Ann Arbor Stages of Patients**

| Grouping | Ⅰ ~ Ⅱ stage | Ⅲ~Ⅳ stage | U-value | *P-*value |
| --- | --- | --- | --- | --- |
| Number of cases | 86 | 98 |  |  |
| Age(year) | 59.0（54.0-68.0） | 61.5（55.0-67.0） | 3917.5 | 0.410 |
| FIB(g/L) | 3.07(2.60-3.83) | 3.17(2.49-4.04) | 4051.0 | 0.737 |
| Albumin(g/L) | 39.9（35.9-43.2） | 37.2（34.1-42.2） | 3357.5 | 0.017 |
| Creatinine(μmol/L) | 64.5（51.8-72.3） | 63.0（49.0-77.5） | 4184.0 | 0.934 |
| Calcium(mmol/L) | 2.26（2.18-2.34） | 2.24（2.13-2.35） | 3697.0 | 0.151 |
| Hb(g/L) | 119.5（109.0-130.0） | 106.5（91.5-122.3） | 2769.5 | 0.000 |
| WBC(10^9/L) | 4.95(3.65-6.32) | 4.35(3.14-5.77) | 3509.5 | 0.051 |
| PLT(10^9/L) | 182.50(131.75-247.25) | 157.50(110.50-216.50) | 3549.0 | 0.065 |
| β2-MG(mg/L) | 2.14(1.78-2.97) | 2.51(1.95-3.79) | 3086.5 | 0.002 |
| LDH(U/L) | 204.0(162.0-250.3) | 221.5(176.5-309.5) | 3384.5 | 0.021 |
| CHI3L1 (ng/mL) | 90.90(49.56-116.71) | 117.79(64.21-212.83) | 3030.0 | 0.001 |

**S6.Clinical Characteristics of 92 Newly Diagnosed Patients with Aggressive Lymphoma**

| norm | CHI3L1 low expression group(n=55) | CHI3L1 high expression group(n=37) | X/U Value | P Value |
| --- | --- | --- | --- | --- |
| Sex, n (%) |  |  | 1.134 | 0.287 |
| Male | 28（30.4） | 23（25.0） |  |  |
| Female | 27（29.3） | 14（15.2） |  |  |
| Ann Arbor stage, n (%) |  |  | 7.966 | 0.005 |
| I-II | 28（30.4） | 8（8.7） |  |  |
| III-IV | 27（29.3） | 29（31.5） |  |  |
| Symptoms, n (%) |  |  | 4.023 | 0.045 |
| With group B symptoms | 21（22.8） | 22（23.9） |  |  |
| No group B symptoms | 34（37.0） | 15（16.3） |  |  |
| IPI score, n (%) |  |  | 9.306 | 0.010 |
| 0-1 | 24（26.1） | 5（5.4） |  |  |
| 2-3 | 21（22.8） | 22（23.9） |  |  |
| 4-5 | 10（10.9） | 10（10.9） |  |  |
| Age | 59.0(52.0-66.0) | 65.0(57.5-73.5) | 634.5 | 0.002 |
| FIB (g/L) | 3.09(2.51-3.79) | 3.48(2.70-4.46) | 757.0 | 0.051 |
| Albumin(g/L) | 38.0(35.5-42.3) | 35.3(32.5-38.8) | 663.0 | 0.005 |
| Creatinine(μmol/L) | 63.0(49.0-71.0) | 73.0(54.5-95.5) | 711.0 | 0.015 |
| Calcium(mmol/L) | 2.28(2.18-2.37) | 2.24(2.13-2.34) | 802.0 | 0.086 |
| Hb(g/L) | 113.0(94.0-123.0) | 106.0(94.0-116.5) | 848.0 | 0.177 |
| WBC(10^9/L) | 4.85(3.55-6.81) | 5.09(4.18-7.52) | 898.5 | 0.343 |
| PLT(10^9/L) | 216.0(157.0-283.0) | 183.0(125.5-296.0) | 882.0 | 0.281 |
| β2-MG(mg/L) | 2.17(1.74-2.93) | 3.60(2.42-5.40) | 409.5 | 0.000 |
| LDH(U/L) | 209.0(169.0-258.0) | 259.0(182.5-396.5) | 701.0 | 0.012 |

**S7.Unifactor COX analysis**

| Variables | β | S.E | Z | *P* | HR (95%CI) |
| --- | --- | --- | --- | --- | --- |
|  |  |  |  |  |  |
| CHI3L1 | 0.004 | 0.001 | 4.576 | <.001 | 1.004 (1.002 ~ 1.005) |
| LDH | 0.001 | 0 | 3.394 | <.001 | 1.001 (1.001 ~ 1.002) |
| β2-MG | 0.14 | 0.052 | 2.688 | 0.007 | 1.150 (1.039 ~ 1.273) |
| Creatinine | 0.006 | 0.002 | 2.336 | 0.02 | 1.006 (1.001 ~ 1.011) |
| HR: Hazard Ratio, CI: Confidence Interval | | | | | |

**S8.** **Calculation of the cut-off value of CHI3L1 by the Youden index**

| CHI3L1/β-actin | Sensitivity% | | 95% CI | Specificity% | 95% CI | Likelihood ratio |
| --- | --- | --- | --- | --- | --- | --- |
| > 0.0002140 | 100 | | 75.75% to 100.0% | 10 | 0.5129% to 40.42% | 1.111 |
| > 0.0003026 | 100 | 75.75% to 100.0% | | 20 | 3.554% to 50.98% | 1.25 |
| > 0.0003945 | 100 | 75.75% to 100.0% | | 30 | 10.78% to 60.32% | 1.429 |
| > 0.0004298 | 100 | 75.75% to 100.0% | | 40 | 16.82% to 68.73% | 1.667 |
| > 0.0004695 | 91.67 | 64.61% to 99.57% | | 40 | 16.82% to 68.73% | 1.528 |
| > 0.0004931 | 91.67 | 64.61% to 99.57% | | 50 | 23.66% to 76.34% | 1.833 |
| > 0.0005308 | 91.67 | 64.61% to 99.57% | | 60 | 31.27% to 83.18% | 2.292 |
| > 0.0005967 | 91.67 | 64.61% to 99.57% | | 70 | 39.68% to 89.22% | 3.056 |
| > 0.0006569 | 91.67 | 64.61% to 99.57% | | 80 | 49.02% to 96.45% | 4.583 |
| > 0.0007021 | 83.33 | 55.20% to 97.04% | | 80 | 49.02% to 96.45% | 4.167 |
| > 0.0007946 | 83.33 | 55.20% to 97.04% | | 90 | 59.58% to 99.49% | 8.333 |
| > 0.0008692 | 83.33 | 55.20% to 97.04% | | 100 | 72.25% to 100.0% | Cut-off value of Youden index calculation |
| > 0.001128 | 75 | 46.77% to 91.11% | | 100 | 72.25% to 100.0% |  |
| > 0.001459 | 66.67 | 39.06% to 86.19% | | 100 | 72.25% to 100.0% |  |
| > 0.001553 | 58.33 | 31.95% to 80.67% | | 100 | 72.25% to 100.0% |  |
| > 0.001669 | 50 | 25.38% to 74.62% | | 100 | 72.25% to 100.0% |  |
| > 0.002325 | 41.67 | 19.33% to 68.05% | | 100 | 72.25% to 100.0% |  |
| > 0.002939 | 33.33 | 13.81% to 60.94% | | 100 | 72.25% to 100.0% |  |
| > 0.003014 | 25 | 8.894% to 53.23% | | 100 | 72.25% to 100.0% |  |
| > 0.003343 | 16.67 | 2.961% to 44.80% | | 100 | 72.25% to 100.0% |  |
| > 0.005734 | 8.333 | 0.4274% to 35.39% | | 100 | 72.25% to 100.0% |  |
|  |  |  | |  |  |  |
